# Multiple Complications in a Sickle Cell Disease Patient: A Case Report

**DOI:** 10.4137/ccrep.s812

**Published:** 2008-07-08

**Authors:** J.A. Olaniyi

**Affiliations:** Department of Haematolgy. University College Hospital, PMB 5116, Ibadan, Nigeria.

## Abstract

Summary

This case report illustrates the multiple complications experienced by a sickle cell anaemia patient. Although he enjoyed fairly good health till age 16 years; he subsequently suffered grade four bilateral femoral head necrosis, cerebella infarct and cerebral atrophy from cumulative effect of repetitive vasoocclussion, recurrent overwhelming septicaemia, fixed flexion deformities and decubitous ulcer as a sequelae of earlier complications. He eventually became bed ridden. Financial constraint seriously compounded these problems. The determinants, of which type of the wide-ranging complications of SCD a particular patient will eventually develop, remain elusive.

## Introduction

Sickle cell disease (SCD) results from the substitution of a valine residue for glutamic acid at position 6 in the beta-subunit of haemoglobin.^1^ Sickle cell disease is a common genetic disorder, which represents a major medical problem in Africa and especially in Nigeria where about 1.2 million are sufferers of the disease.^2^ It is characterized by chronic haemolytic anaemia, vasoocclusive process and multiple organ infarction resulting from wide spread vascular occlusion.

Deoxygenated Hb S tends to polymerize non-covalently into long strands that deform the erythrocyte, giving the characteristic “sickle cell” morphology^3^ whereas Hb S with bound oxygen in the arterial circulation for example, does not polymerize. The most widely accepted hypothesis is that erythrocytes deform as they release their oxygen in the capillaries and are trapped in the microcirculation.^4^,^5^ The blockade of blood flow produces areas of tissue ischemia, leading to the myriad of clinical problems seen with sickle cell disease. Other pathophysiologic mechanisms include slugging resulting from increased viscosity, abnormal adhesion of red cells to endothelial cells, microvascular occlusion and constant vascular damage.^3^,^5^ Also, involvement of multiple components of the blood like accompanying leukocytosis and thrombocytosis equally contribute to disturbed blood flow.^6^ The rate at which red blood cells undergo sickling in deoxygenated state, which partially determine the clinical course; is highest in homozygous sickle cell anaemia ( HbSS), followed by Hb SC, Hb SD and by Hb S-beta thalassaemia.^7^,^8^ Again, the great variability in severity of sickle cell disease is governed by various other genetic determinants and environmental factors.^9^,^10^ SCD, once more, is extremely varied in its manifestations in terms of both the organ systems that are affected as well as the severity of the affliction.^11^,^12^ However, the determinants of which complication(s) a particular SCD patient will develop remain elusive. Hence, there is a great need for close monitoring and counselling of all patients in order to avoid preventable complications.

## Case Report

This 31 year old male by initials O.K. a son of a low cadre soldier (retired), was first diagnosed at age 10 years at the University College hospital, Ibadan and was followed up by the Paediatricians until age 16 years when he was taken over by the Haematologists of the same hospital. These early years were uneventful.

In 1987, however, he was diagnosed of grade 4 avascular femoral head necrosis by the Orthopaedic surgeons and he was advised to be on crutches. Thereafter, he defaulted in his clinic attendance.

In July 2005, he presented with generalised bone pains and persistent fever of 8 days duration. There was associated headache and inability to sleep. Examination showed that he was in moderately severe painful distress, moderately dehydrated, pale but afebrile, anicteric, and acyanosed. There was no significant peripheral lymphadenopathy. He was tachypnoiec ( respiratory rate of 40 cycles per minute) but the breath sounds were globally vesicular. The pulse rate was 90 beats per minute, BP 120/70 mm Hg, normal heart sounds 1 and 2 were present. The abdomen was full, tense and with some degree of guarding. The musculoskeletal system examination revealed diffuse tenderness of the legs, thighs and the arms. The overall impression on admission was vasoocclusive crisis in a HbS patient and was handled as such. However, in the course of treatment, patient suddenly developed tonic clonic seizure for the first time in his life time and it was generalized and lasted 60 seconds. This gave us an impression of CNS event? infarction. He was transfused with a unit of packed cells because of a haematocrit of 18%, he had a week course of parenteral ciprotab and flagyl in view of tense, tender abdomen suggestive of acute abdomen [?peritonitis; ?? omental infarct]. He was also on nil per oral until the abdominal tenderness resolved. He was discharged after one week of hospital admission.

Patient represented with high grade fever and generalized bone pains nine months after discharge, He later became confused and was talking irrationally. In addition to this, patient was tremulous and was having occasional jerky movement of the limbs. Patient was presumptuously treated for meningitis with ceftrazone 1 g every 12 hrs in view of the fact that observation is said not to be a good option when a SCD patient is suspected to have septicaemia. Eventually blood culture report yielded klebsiela species, sensitive to sperfloxacine, ciprofloxacin and perfloxacine. He was treated with ciprofloxacine. The neurologists reviewed in view of the left spastic hemiparesis which had developed and agreed with the impression of septicaemic illness. They then added artane, diazepam and neurobion tablets to the management in order to control the tremulousness. It took the patient about four weeks to do the cranial CT scan requested for by the neurologists because of financial constraints. The outcome of delayed cranial CT scan showed cerebella infarction and cerebral atrophy. There was no remarkable improvement in the condition of the patient in spite of treatment so far instituted in the two months of re-admission. Our efforts to further review the patient for the underlying cause was truncated by the patients request for discharge against medical advice because of serious financial constraint.

However, 4 months after patient had gone home against medical advice, he represented to our haematology day care Unit in a very bad and pathetic clinical state. He had developed severe muscular atrophy and fixed flexion deformity of both knees, multiple bed sores on the right buttocks and the knee. He was unable to walk, chronically ill-looking and febrile. The febrile illness was treated this time with chloramphenicol and floxapen for 6 weeks pending the outcome of blood culture. Temperature settled eventually. Pressure sores were dressed daily with honey and the physiotherapists were invited. The orthopaedic surgeons were finally invited in view of a repeat x-ray of both hip joints showing grade four femoral head necrosis of both hip joints. Although he remained incapacitated, he was fairly well in bed for the period of about three month’s admission.

The final effort to rehabilitate him by getting him a wheel chair because of his inability to afford hip replacement, has not succeeded yet in spite of efforts put in by the social workers to raise fund for him. He was constrained to go home in that incapacitated state and remained lost to follow up.

## Discussion

This case report simply highlights the agony a sickle cell disease patient without sufficient financial and social support experiences in the developing country economy in the course of the disease. It has been noted that complications in SCD is extremely varied^11^,^12^ both in the organ systems that are affected as well as the severity of the affliction. Platt et al. 1991 noted that while a significant number of patients with the disease have few admissions and live productive and relatively healthy lives; about 5% of patients account for nearly one third of hospital admissions.^9^ This index case contributes to the 5% in view of multiple and prolonged hospital admissions.

Complications in SCD arise as a consequence of three main pathophysiological mechanisms i.e. Vasooclussive [VOC] complications, hyper-haemolytic complications and infective complications. The VOC complications include stroke, acute chest syndrome, priapism, splenic sequestration, liver diseases, leg ulcers, osteomyelitis, retinopathy, renal insufficiency. Haemolytic complications include anaemia, cholilithiasis and aplastic and megaloblastic crises. Infective complications could result from viral, bacteria, fungal and atypical microorganisms but common ones include encapsulated organisms like Strept. pneumoniae infections, bacteria causing chronic osteomyelitis and E. coli sepsis especially in children.

Presence or absence of these chronic complications notwithstanding; the disease is often interrupted (depending on severity of the disease) by crisis which is usually infarctive, but can be hyper-haemolytic, aplastic or megaloblastic crisis.

Irreversible, progressive and eventually debilitating and incapacitating complications like stroke, advanced femoral head necrosis, chronic renal failure etc constitute a huge financial budding and consume the time and energy of the care giver(s). In this patient, prominent complications include radiologically confirmed grade 4 bilateral avascular necrosis of the femoral head, stroke with CT confirmed cerebella infarction and cerebral atrophy and recurrent overwhelming sepsis, culture growing klebsiela species. He also developed decubitus ulcers, severe muscle wasting and fixed flexion deformity.

SCD patients are highly susceptible to overwhelming infection.^13^,^14^,^15^ Splenic autoinfarction during childhood is regarded as the most significant factor.^16^ Another important factor among others is lazy leukocyte syndrome.^16^ Overwhelming sepsis is a frequent cause of hospital admissions by precipitating any of any of the crisis in the SCD patient. This index patient presented with features of infarctive crisis. The implication of this is that, as a rule, the precipitating cause of all infarctive crisis or any other crisis must be clearly established and properly managed in order to fully control the painful episode.

Neurologic complications occur in 25% of HbSS patients. It could be in the form of silent or overt cerebral infarction, Transient ischaemic attack [TIA], cerebral haemorrhage or seizure. Studies have shown that the mechanism involed include narrowing of arteries near sharp turnings and at the separation of middle cerebral and internal carotid arteriesand the arterial narrowing contributes to higher rate of blood flow and this is believed to contribute to the risk of arterial occlusion.^17^,^18^,^19^ It is known to be commoner in children [average age of 4 years] than in adults.^20^ It has been noted that 10% of children develop symptomatic CVA and ishaemic infarction is the most common in age 2–9 years. In adults however, especially in age 20–29 years; haemorrhage complications, most commonly subarachnoid haemorrhage, occur more frequently.^21^ This patient had initial persistent headache, later he had short clonic tonic seizure. He became tremulous in the subsequent admission and finally developed spastic paraplegia. This may suggest that this patient had silent cerebral infarction which later became overt. Studies carried out using MRI 22,23 showed that 20% of SCD patients had suffered silent cerebral infarction. The financial incapacitation of the patient did not allow for proper and adequate investigation to detect the long suspected CVA.

Avascular necrosis of the femoral head (ANFH) is a well documented complication of homozygous sickle cell (SS) disease which may cause considerable morbidity because of persistent pain and limitation of movement. Avascular femoral head necrosis often develops insidiously. It starts by recurrent pain of the hip with occasional acute exacerbations. Pain later becomes more sustained as avascular necrosis advances, leading to limping gait and later dislocations of the hip may occur. It occurs most commonly in adolescence, femoral head necrosis with articular surface disruption affects 10% to 12% of SS patients of African origin.^24^,^25^ Reported haematological risk factors in patients of African origin, include a high total hemoglobin and low fetal hemoglobin (in males only)^26^ and homozygous alpha thalassemia.^24^, ^27^, ^28^ Patients with sickle cell disease should be asked about this symptom at routine clinics and be reminded to report hip pain when it occurs. Main finding on physical examination may be isolated to resistance to rotation of the leg when the patient is lying supine. Plain hip films will detect advanced disease but may not detect early stage disease which can be detected by MRI.^29^

As it was observed in this patient, the activities of the patient with such incapacitating complications is impaired and the caregiver bears all the responsibilities of meeting the patients personal and health needs. The family care caregivers are often overwhelmed not only by the financial burden but also by the stress of meeting the daily needs of such incapacitated patients. It is therefore very obvious that the care of these patients can not be left to the parents or care givers alone. The Government needs to assist these patients in many ways. In the first instance, all SCD patients could be registered by the local authority so as to be able to make adequate budgetary allocations for them. Their hospital care should be shouldered by the Government, just as it is being done for HIV/AIDS patient of recent. If each patient has unlimited assess to good and free health care, occurrence of serious complications will be greatly reduced because majority could have been stemmed in the bud. When these debilitating complications unavoidably set in, the Government should have a rehabilitating centre which should be able to provide all that is needed to make them not just comfortable, but enough to allow them make progress in life. In the case of this patient, he required wheel chair to improve his quality of living in his present circumstance but this remains a Herculean task.
